# Sustainable Accessibility: A Mobile App for Helping People with Disabilities to Search Accessible Shops

**DOI:** 10.3390/ijerph16040620

**Published:** 2019-02-20

**Authors:** Diego Mayordomo-Martínez, Juan M. Carrillo-de-Gea, Ginés García-Mateos, José A. García-Berná, José Luis Fernández-Alemán, Saúl Rosero-López, Salvador Parada-Sarabia, Manuel García-Hernández

**Affiliations:** 1 Department of Structures and Construction, Technical University of Cartagena, 30202 Cartagena (Murcia), Spain; diego.mayordomo@upct.es; 2 Department of Computer Science and Systems, University of Murcia, 30100 Murcia, Spain; jmcdg1@um.es (J.M.C.-d.-G.); josealberto.garcia1@um.es (J.A.G.-B.); aleman@um.es (J.L.F.-A.); saul.rosero@um.es (S.R.-L.); 3 Federación de Asociaciones Murcianas de Personas con Discapacidad Física y Orgánica, 30005 Murcia, Spain; spsarabia@gmail.com (S.P.-S.); accesibilidadfamdif@gmail.com (M.G.-H.)

**Keywords:** accessibility, sustainable app development, motor disability, new technologies

## Abstract

People with motor disabilities must face many barriers and obstacles in their daily lives, making it difficult to perform everyday tasks. The purpose of this work is to improve their living conditions by providing an app with accessibility information in an updated, reliable and friendly form. The development of the system integrates national and regional accessibility regulations, architectural aspects, with an extensive field work, and a sustainable software process. The levels of accessibility and the requirements of the application are defined in the first phases of the project. The field work included the evaluation of 357 commercial establishments in the city of Murcia, Spain, showing that only 25% have a good accessibility, 40% are practicable with help, and 35% are inaccessible shops. The proposed system achieves its objectives of being sustainable and helping in the accessibility. Besides, the system can be a great incentive for businesses to improve their accessibility conditions. In conclusion, new technologies must have a much more active role in the promotion of universal accessibility. These tools must also consider the necessary requirements of sustainable development.

## 1. Introduction

According to the United Nations, around 650 million people, or 10% of the world’s population, live with a disability [1]. What is more, the most vulnerable social groups or minorities usually suffer from higher rates of disability, such as the people with lower incomes, the people with lower levels of education, the elderly, women and street youth, which make them even more disadvantaged. According to the Spanish National Institute of Statistics (INE) in 2008, the people with disabilities in the Region of Murcia, where this research has taken place, represent a 9.8% of the whole population. Moreover, the report “Disabilities in the Region of Murcia. Territorial and temporal distribution 2000–2012”, of December 2012 [2], indicated that there were 172,209 people with disabilities, which represents 11.8% of the inhabitants of the Region. These figures are in line with those provided by the international organisms mentioned before. In addition, the most prevalent form of disability in Spain for both men (4.3%) and women (7.7%) is related to mobility [3].

People with physical disabilities often encounter insurmountable obstacles in their daily lives. This can prevent them from doing things that would be normal for anyone, such as having access to leisure and cultural activities, finding a job, buying in a shop, visiting public buildings, using public transport and even leaving their own homes. 

The International Organization for Standardization (ISO) and the International Electrotechnical Commission (IEC) define accessibility as the “extent to which products, systems, services, environments and facilities can be used by people from a population with the widest range of characteristics and capabilities to achieve a specified goal in a specified context of use” [4]. International standardization bodies thus focus on accessibility from an inclusive perspective, and consider that accessibility generally benefits everyone, not just people who suffer from some type of disability.

The United Nations (UN) Convention on the Rights of Persons with Disabilities [5] (CRPD) provides in Article 9 an international obligation for States Parties to take appropriate measures to ensure accessibility to the physical environment. Particularly, Point 2(b) establishes that States should take measures to ensure that private entities open to the public “take into account all aspects of accessibility for persons with disabilities”. On the other hand, the UN Sustainable Development Goals [6] (SDGs) are to address all three dimensions of sustainable development (environmental, economic and social) in order to mitigate global problems such as poverty, inequality, climate, etc. Disability is included in the SDGs, in particular as regards accessibility of human settlements: “Goal 11—Make cities inclusive, safe, resilient and sustainable” encourages Member States to “provide universal access to safe, inclusive and accessible, green and public spaces, in particular for (...) people with disabilities”. Moreover, in the UNs Conference on Housing and Sustainable Urban Development, world leaders adopted the New Urban Agenda (http://habitat3.org/the-new-urban-agenda/) and established the new global standards in sustainable urban development; to achieve its goals, cooperation between the governments, civil society and private sector is required.

According to [7], “sustainable accessibility” is defined as accessibility “with as little as possible use of non-renewable, or difficult to renew, resources, including land and infrastructure”. This concept is often used in the context of the urban planning and transport literature [7,8,9]. It is connected with the idea of making efficient use of resources, and it is achieved by identifying solutions where economic, social, and environmental goals could be combined. Sustainability or sustainable development rests on these three pillars—i.e., economic, social, and environmental—which are all equally important [10].

Following these goals of accessibility and sustainability, the objective of the present research is to create a new tool to help people with motor disabilities by providing information about access to the public establishments in the area of study. This area is located in the city of Murcia, Spain, a medium-sized city of around 400,000 inhabitants that is the capital of the Region of Murcia. This is an interdisciplinary research that has counted on the contribution of computer science engineers, architecture specialists, and the regional organization FAMDIF (Federation of regional associations of people with physical and organic disabilities). While *physical* (or motor) *disability* is well known to be the limitation in doing certain movements, the term *organic disability* is less common. It refers to people with some damaged internal organ, often associated with non-perceptible diseases. For example, cystic fibrosis, Crohn's disease, hemophilia, hepatitis and diabetes are some of the diseases that can lead to organic disability. The present research is based on extensive fieldwork carried out in the city of Murcia, where more than 357 shops, banks, restaurants, etc., were analyzed. This is, to the best of our knowledge, the broadest study done on public establishments at a national level.

In general, many projects about technological solutions of accessibility for people with disabilities can be found, although very little scientific papers have been published so far. For example, in the case of Spain, the national organization Spanish Confederation of People with Physical and Organic Disabilities (COCEMFE) has promoted the development of several apps for their members. We can mention the app *Accessibility Plus* (http://www.fundacionvodafone.es/apps-accesibles/linea-accesibilidad), which is intended to locate the nearest accessible taxi and consult more than 41,600 points of interest free of barriers in Spain, and the recent project *Mapcesible* (https://mapcesible.fundaciontelefonica.com) a collaborative system where the users can introduce information of the places they visit, and currently has more than 22,000 evaluations. Also, the app called *Línea accesibilidad* (http://www.fundacionvodafone.es/apps-accesibles/linea-accesibilidad) has been created to notify the local authorities about the problems of accessibility detected by the users. Another interesting project is the app *TUR4all* (https://www.tur4all.com), which pursues that people with disabilities can travel, enjoy touristic resources and participate in the leisure activities as any tourist. This app publishes the touristic resources analyzed by experts and included by the users through a questionnaire of evaluation. Also, in the context of Spain, *SIMON Mobile* is an app supported by the Assisted Mobility for Older and Impaired Users (SIMON) project and funded by the European Commission. This app was designed so that people with reduced mobility could find parking spaces, calculate routes, and locate lifts or ramps in public transport systems in Madrid, Lisbon and Parma. *Turismo Accesible* app (https://www.equalitasvitae.com/es/servicios/app.php) by Equalitas, collects data and classifies tourist services (hotels, routes, restaurants and museums) according to their adaptability. Other examples are *VENZE* (https://venze.es/), an app which allows people with reduced mobility to travel in accessible vehicles, and *Disabled Park* (https://www.disabledpark.com/), a web platform and mobile application to locate parking spaces on a map and report those cars that are using improperly spaces reserved for people with disabilities.

In other countries, some developments are addressed for specific locations such as: (1) *Access GC* (https://play.google.com/store/apps/details?id=au.edu.griffith.accessgc2), an app created by the Griffith University to give information about access to tourist resources in the Gold Coast of Australia; (2) *Access Aware* (https://play.google.com/store/apps/details?id=com.thundermaps.ccs), an app designed to provide information and feedback about parking for people with disabilities in New Zealand; and (3) *Access4All* (https://access4allproject.eu), an app to offer accessible and inclusive venues and services on Far South Coast of New South Wales.

On a wider level, many other accessibility apps can be found in the most popular markets, such as Google Play and Apple Store. For example, *Access Earth* (https://access.earth) is an ambitious project that tries to include different aspects of accessibility such as parking, bathrooms, buildings, etc., in a free and global community. Similarly, *AccessNow* (http://accessnow.me) provides an app and tries to create a global community to search and share accessibility information. Also, *AccessAble* (https://www.accessable.co.uk) is a project that offers an app to provide accessibility of more than 10,000 venues, mainly in England and Ireland.

As can be seen, in the applications with a greater geographic scope, the information is normally provided by the user, while the projects for small areas are usually based on technical field works. In any case, the lack of scientific publications is evident, as has already been mentioned.

The environmental and social dimensions of sustainability are specifically addressed in the present article. Moving onto the solution space, the difference between “sustainable in software” and “sustainable by software” can be highlighted [11]: the former refers to whether the mobile application itself is sustainable, whereas the latter points out at whether using the mobile application has a positive effect on sustainability. The mobile application developed has a potential impact on both areas: (i) “sustainable in software”; a sustainable process was followed to develop the software, and the product is energy-efficient (i.e., the mobile application targets the environmental dimension of sustainable development); and also (ii) “sustainable by software”; the purpose of the software is to help people with motor disabilities (i.e., the mobile application focuses on the social dimension of sustainability).

The rest of the paper is organized as follows: Section 2 describes the technical definition of the levels of accessibility and the field research that was carried out. Section 3 presents the software development process. Then, the experimental results are given in Section 4, including a running example and the evaluation of usability of the app. These results are discussed in Section 5, comparing our proposed with other existing projects. Finally, the main conclusions are drawn in Section 6.

## 2. Materials and Methods 

### 2.1. Definition of the Accessibility Levels

Unfortunately, there is no universally accepted standard for the definition of the levels of accessibility to buildings. In fact, since the parameters of interest—such as the length, slope and height of the ramp, or the shape and height of the step—can take infinite values on a continuous scale, people with different motor disabilities could have varied opinions about these criteria. For example, according to the type of wheelchair (manual or electrical), the size of the wheels, and the strength and skill of the user, the values of the maximum allowed ramp or step can be very different.

Thus, the definition of the parameters that define the accessibility levels is an essential preliminary step. Basically, two points of view can be considered: the regulation established in national and regional laws; and the opinion of the users obtained through their representatives. In our case, the regulation is provided by the Spanish Code of Building, section Basic Document of Security of Use and Accessibility [12]. This code defines a building as accessible when it can be entered without any step, at street level, or by means of a slanting plane (or ramp) with a maximum slope of 4%.

The intermediate level of accessibility given in the cited Spanish regulation is composed by entrances with ramps that are considered easy to climb up. In general, higher slopes are allowed for shorter ramps, and lower slopes for longer ramps. More specifically, the following possibilities are allowed: a ramp up to 3 m long with up to 12% slope; a ramp up to 10 m long with up to 10% slope; a ramp up to 15 m long with up to 8% slope; a ramp of up to 6% slope, without any limit of length; a step up to 5 cm high with a ramp up to 25% slope. These parameters are included in the Spanish regulation for existing buildings [13]. In addition, the local regulation of the area of study includes in this intermediate level of accessibility two more possibilities: buildings with a step up to 3 cm high with a rounded border; and steps up to 12 cm high with a ramp up to 30% slope. These parameters are defined in Order of 15th October 1991 [14]. Finally, according to these national and regional rules, the rest of buildings are considered as not accessible.

However, the accessibility experience of the users can be far from the requirements established by the existing laws. For example, a slope of 30% can be impracticable for most electric wheelchairs, even if the length of the ramp is only 12 cm. Therefore, we have collected and analyzed the experience of the users in practice, through a series of meetings held with the representatives of the regional association FAMDIF. This association is one of the most important at a regional level, and currently has more than 3000 members and gives services to all the people with physical and organic disabilities. Considering the aspects of practicality, usability and simplicity, it was decided to classify accessibility into four levels, named with uppercase letters, A, B, C and D, from more to less accessible. The criteria and parameters that define each group are:

- *Level A*. This level corresponds to accessible buildings. This category includes entrances at street level without any obstacles. Small obstacles that can be easily overcome by all users are also tolerated: a step with a maximum of 1 cm, or a ramp with a slope up to 8%.
- *Level B*. This level contains the buildings which are accessible with a low or moderate difficulty by most users. This situation occurs when the access is done through a ramp with a slope of 10% or less. A maximum slope of 25% is allowed for small heights of 5 cm or less.
- *Level C*. This level refers to the buildings whose entry is practicable with the help of other people. According to the experience of the users, some help is needed when the ramps have a slope greater than 10% and up to 12%, or a step up to 12 cm in height. This level includes steps up to 12 cm with a ramp of less than 30% slope. Finally, this category also includes entries with a step of less than 3 cm of height, but with a rounded border.
- *Level D*. The rest of the cases are included in this level D, which contains all the buildings which are considered as not accessible. This means that buildings with a ramp of more than 12% slope or a step higher than 12 cm are not accessible by people with reduced mobility under common circumstances.

Table 1 summarizes the four levels of accessibility defined and their main parameters. Observe that each level receives a descriptive name: accessible; accessible with difficulty; practicable with help; and not accessible.

### 2.2. Collection of Accessibility Information

**Figure 1:**
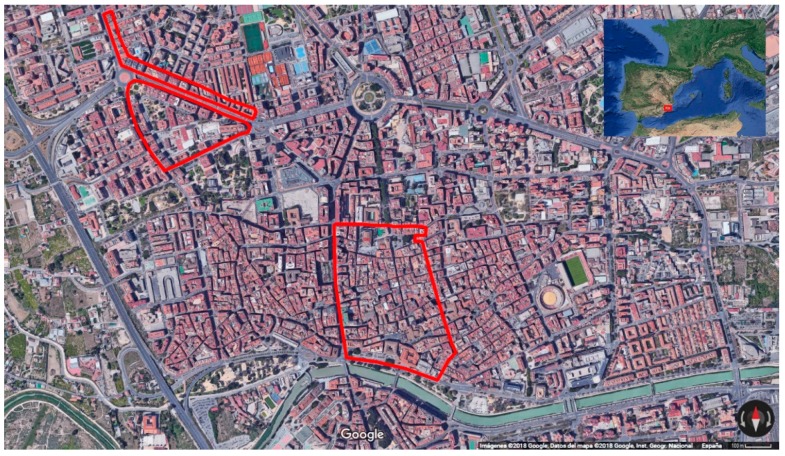
Satellite image of the city of Murcia, Spain. The area studied in the fieldwork is marked in red. Source: Google Earth.

After defining the different levels of accessibility, an extensive fieldwork was carried out in the center of the city of Murcia to collect data from shops, banks, bars, restaurants and other public establishments. A total of 357 buildings belonging to the quarters of Historic Centre, Saint Anton and Saint Maria of Grace were visited and checked, covering around a 10 % of the city. Figure 1 shows a map of the studied area.

**Figure 2:**
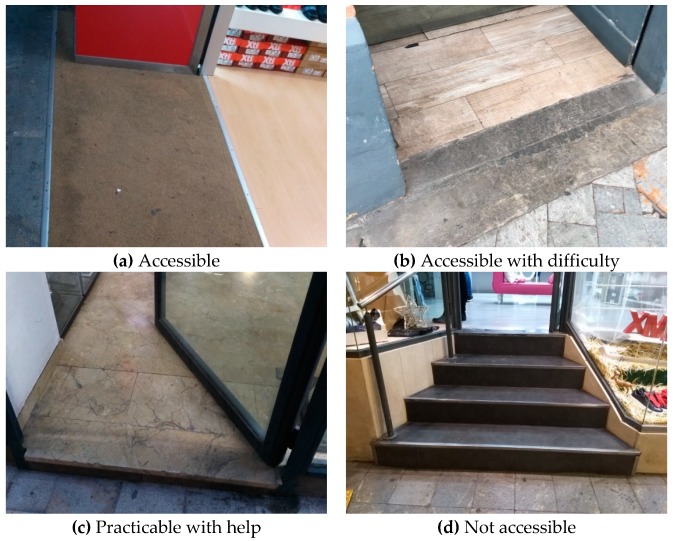
Samples of the four levels of accessibility found in the field study. (**a**) A store with accessible entrance. (**b**) An entrance accessible with some difficulty. (**c**) An entrance that can be accessed with the help of other people. (**d**) A not accessible entrance.

The field study was conducted with the help of the technical staff of FAMDIF and the assistance of the researchers of this work. The process to obtain the information was the following:

- Analysis and distribution of the geographical areas of the zone under study, and schedule of visits to the establishments. The study took place over a period of 8 months, from December 2017 to July 2018, and it involved three technicians.
- Visit to the shops to check out their accessibility. The technical staff measured parameters such as the number and height of the steps, the length and slope of the ramps, the width of the entrance, and the type of door handle. Other parameters of interest, that are indicated in the following sections, such as the name of the shop, the type of activity, and the GPS location, were also collected. Photographs were taken of the public part of the buildings, avoiding the appearance of people.
- Evaluation of the level of accessibility to the buildings, according to the classification previously defined and shown in Table 1. Some sample pictures of each category are shown in Figure 2.
- Preparation of a summary table showing the levels of accessibility of all the establishments studied, indicating the characteristics of the entrance, the type of door, whether or not it has stairs, ramps, and any other information which can be relevant for people with disabilities. This table also contains the geographical location of the buildings, using GPS coordinates, the type and subtype of the establishment, and the date of the last visit. This information is used to create the database of the app.

The results of this field work show that there are 35% inaccessible shops (level D), 40% practicable with help (level C), 5% accessible but with some difficulty (level B), and 20% with high accessibility (level A). This means that only one in four establishments is friendly for people with disabilities (levels A and B). Most buildings (40%) include accessibility measures but they are not enough (level C). These results are in line with those obtained in other national studies. For example, in [15] a study about the accessibility to housing buildings in Spain is presented. This work includes 1211 telephone surveys, but only 43 detailed technical inspections. It concluded that only 36% of the buildings can be accessed without steps or ramps. Moreover, considering the entrances with ramps, only 26.8% meet all accessibility criteria. On the other hand, the recent report [16] describes an accessibility study to buildings in Spain with 2027 surveys. They concluded that 63% of the buildings are inaccessible because they have steps in the entrance. Only 28% of the total have a ramp, but in most cases it is not practicable.

## 3. A Sustainable Mobile Application for Finding Accessible Shops

The purpose of this research is to help people who suffer from some type of motor disability to cope better with their urban environment and to avoid some of the day-to-day difficulties derived from their condition. To develop the software solution, a hybrid approach was applied in our project, mixing techniques together to fit our needs: a detailed requirements effort was carried out, followed by many implementation iterations and increments in the app functionality. A user-centered design process was also applied in this work. The engineering team: (1) worked closely with stakeholders from FAMDIF to outline the context of use of the mobile app; (2) documented the software requirements; (3) proposed a design solution; and (4) evaluated it against the requirements baseline for quality evaluation (verification). An iterative process was followed to evolve our app and ensure that it met the stakeholders’ expectations. In addition, a usability audit (validation) was carried out by applying a heuristic evaluation approach.

### 3.1. Analysis and Selection of Technological Alternatives

The massive adoption of mobile devices by the general population makes this type of hardware a good option to reach most people. A mobile application was thus chosen to achieve our goals. *Android*, the mobile operating system developed by Google, is the most popular operating system worldwide. According to Gartner, market share of Android devices accounted for 85.9% of all smartphone sales to end users in 1Q18 [17]. For this reason, Android was the platform of choice for developing the software. It is advisable to use an Integrated Development Environment (IDE) to improve efficiency and facilitate the implementation of software applications. *Android Studio*, based on JetBrains’ IntelliJ IDEA, is the official IDE to create apps for Android. In particular, Android Studio V. 3.1.0 (Google LLC, Mountain View, CA, USA) was used in this project.

The required behavior of the mobile application (see Section 3.2) made it also necessary to use several libraries and tools. MySQL is the world’s most popular open source database, especially for web development projects. Although Oracle (Oracle Database) and Microsoft (Microsoft SQL Server) database solutions rival MySQL in terms of popularity, MySQL V. 5.0.12 (MySQL AB, Cupertino, CA, USA) was chosen as a free and open source solution. The programming language selected was PHP (V. 7.2.5) because it is free software and especially suited to web development. It allows executing the code on the server side, making queries to the database and returning the result to the user. Google Play Services V. 11.8.0 (Google LLC, Mountain View, CA, USA) offer Google-powered features and automatic updates to Google apps and Google Play apps; this component was also included in the project, since it provides functionality such as energy-efficient services based on location. Retrofit V. 2.1.0 (Square Inc., San Francisco, CA, USA) is a REST client for Android, developed by Square, which allows making HTTP requests, managing different types of parameters, and automatically parsing the outcome to a Plain Old Java Object (POJO).

Other libraries were specifically used to implement the communication between the mobile device and the server. Volley is a library developed by Google to optimize the dispatch of HTTP requests from the Android applications to the external servers. AsyncTask is another library, developed by Square Inc., which was used for managing tasks and background processes.

### 3.2. Requirements Specification

The features—and requirements—of the mobile application were elicited and documented. A requirement is a “statement which translates or expresses a need and its associated constraints and conditions” [18]. The Software Requirements Specification (SRS) comprises the requirements agreed with the stakeholders, and represents a baseline to start building the software product. Moreover, features are requirements stated at a high level of abstraction [19]. The SRS was organized according to the ISO/IEC/IEEE 29148:2011 standard, thus taking advantage of our previous experience [20,21]. An excerpt of the SRS that includes some relevant requirements is as follows:

- *SRS1*. The application shall display the following details of the establishments: name, address, GPS coordinates, type, subtype, accessibility level, the description of the entries, specific description of the doors and ramps in the access to the shop, the opening time, toilet, comments, the date of registration and the last modification date.
- *SRS2*. The establishments are divided into types and subtypes. Possible types are: food; restaurant industry; education; health; aesthetics and personal care; textile and accessories; leisure and entertainment; banks, insurance and management; real estate; home and decoration; technology; automotive; others. Each type group has its own subtype classification.
- *SRS3*. The application shall provide the following actions: search an establishment, sign in, log in, send suggestions, view user’s suggestions, rate establishments, manage establishments, and delete users.
- *SRS4*. There are three kinds of roles in the application, depending on the type of user: unregistered user, registered user and administrator. Each of them has different actions available:
  o *SRS4.1*. Unregistered users can search a shop for further information, sign in the community of users to become a registered user, and log in the app through an account previously created.
  o *SRS4.2*. Registered users can do all unregistered user actions. In addition, they are able to send suggestions, and rate the shops according to the characteristics of the accesses.
  o *SRS4.3*. Administrators can do all registered user actions. They can also manage the database from the mobile application. Moreover, they can view user’s suggestions for improving the application, manage the information stored about the shops to update it if needed, and remove malicious users.
- *SRS5*. The application shall show the location of the establishments on a map, and allow the user to select them directly from the map results.
- *SRS6*. The application shall allow the users to rate the accessibility of the establishments from 1 to 5, and they will have an average rating depending on the scores received.

On the other hand, there are also some design constraints. A design constraint (DS) is “a requirement that limits the options open to a designer of a solution by imposing immovable boundaries and limits” [18]. The DS that were documented in the SRS are:

- *DC1*. An embedded third-party application shall allow us to locate the establishments searched and calculate their distance.
- *DC2*. The architecture of the application shall be designed considering connections from multiple Android clients with a centralized server.
- *DC3*. The application shall connect to a remote server located in FAMDIF where the data will be stored.
- *DC4*. The application shall minimize the number of queries to the server to save mobile data and battery.

**Figure 3:**
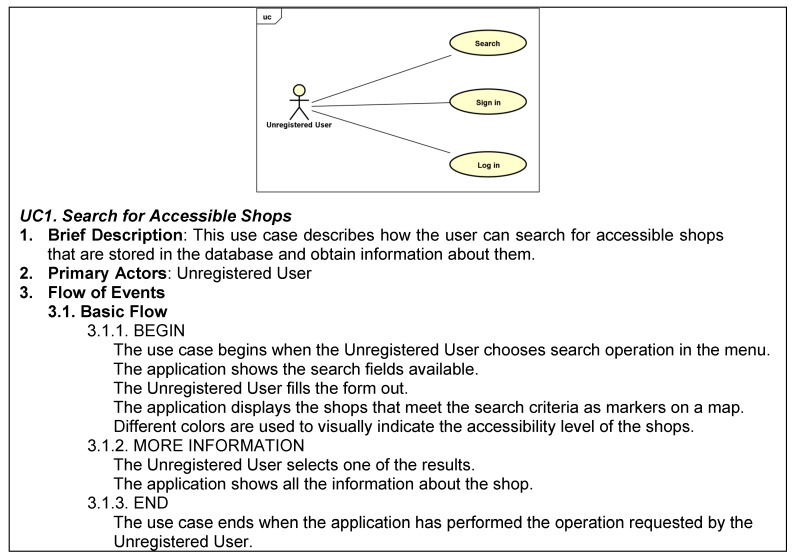
Use case diagram for the unregistered user.

Use cases were also documented as another mean of capturing requirements. A use case defines interactions between actors and the system to achieve a goal [22]. The use case presented in Figure 3 is especially relevant, since it illustrates the search for accessible shops. More details on this key functionality of the application are presented in Section 4.

### 3.3. Design of the App

The design of the developed app follows the standard model-view-controller architecture; the model defines the data structure of the information stored in the system, the view defines the user interfaces, and the controller contains the control logic of the application. Fulfilling the requirements of the app, the model consists of different POJOs which are used by the Volley library. These are objects composed by primitive Java types, which the library automatically builds from the decoding of the JSON sent by the PHP in the server. This way, the objects exchanged between the server and the phone are as small as possible, in order to save the consumption of data.

**Figure 4:**
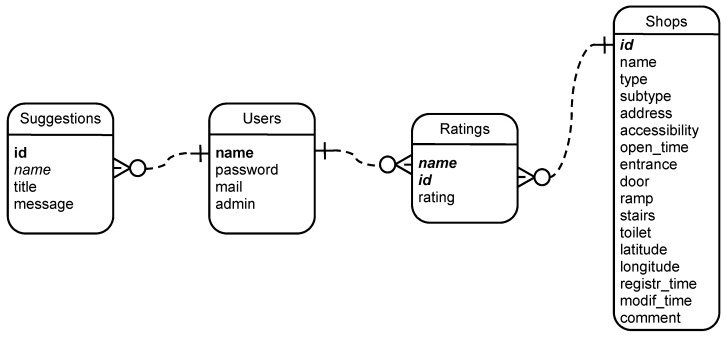
Entity-relationship diagram of the information stored in the database.

The design and creation of the tables of the database, located in the server, is derived from the entity-relationship diagram shown in Figure 4.

It can be observed that the database consists of four entities:

- *Shops*. This is the main entity of interest of the system. They are identified by a unique identifier and contain all the attributes defined in Section 3.2. Some of them are required (such as the name, type, subtype, address, accessibility level, GPS coordinates, and date of the last visit), and the rest are optional (such as the opening time, entrance, door, ramp, stairs, toilet and comments).
- *Users*. The second main element of the system are the users. A minimal set of attributes is stored to comply with data protection legislation. This includes a unique nickname, a password, and an e-mail address which must be unique. A Boolean attribute “admin” indicates if the user is an administrator or a normal user.
- *Ratings*. Registered users are able to rate the shops that they have visited, according to their accessibility experience. Thus, a rate is defined by the unique name of the user, the id of the shop and the rate, from 0 to 5. In this way, a user can only vote once for each shop.
- *Suggestions*. Apart from the rating, registered users can make suggestions, which are addressed to the administrators of the system. They are not associated to particular shops, because the user can make a general suggestion. They have a unique id, a title, a message, and the name of the user.

**Figure 5:**
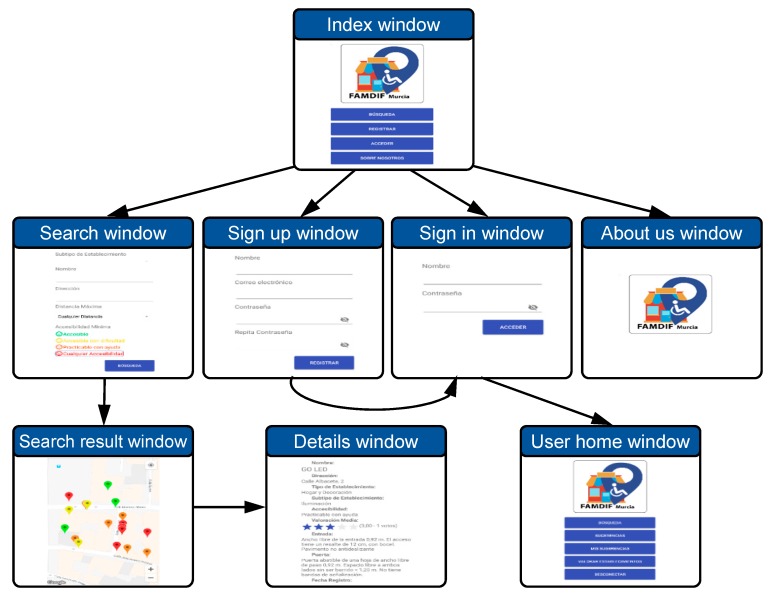
Navigation diagram of the app, corresponding to the role of unregistered user.

On the other hand, the development of the view is guided by the navigation diagram. It contains the functionality for the different roles: unregistered user, registered user, and administrator. As an example, Figure 5 presents the part of the navigation diagram corresponding the unregistered user, which is used since the app is started until the user logs in.

The first element is the *index window*, which is shown when the app is started. It contains access to the actions available for a not registered user. The main functionality is given by the *search window*. The search is defined by a set of filters by name, type and subtype of activity, proximity and level of accessibility. All of them are optional. After clicking in the search button, the *search results window* is opened showing a map of the resulting establishments. They are marked with a color (green, yellow, orange and red) corresponding to the accessibility level (A, B, C and D, respectively). Clicking in the market, the *details window* displays the information about that shop. This information includes the average rating of that shop. Other operations accessible from the index window are the *sign up window*, to create a new user account, the *sign in window*, to enter the advanced mode of operation, and the *about us window*.

The role of registered user includes these functionalities plus two new options: rating an existing establishment, and proposing a new suggestion; both with the corresponding windows. The role of administrator user has additional operations to edit, add and remove establishments, and to manage suggestions and users. In this way, the app is prepared to allow a complete administration of the system.

## 4. Results

### 4.1. Running Example: Sustainable Search

**Figure 6:**
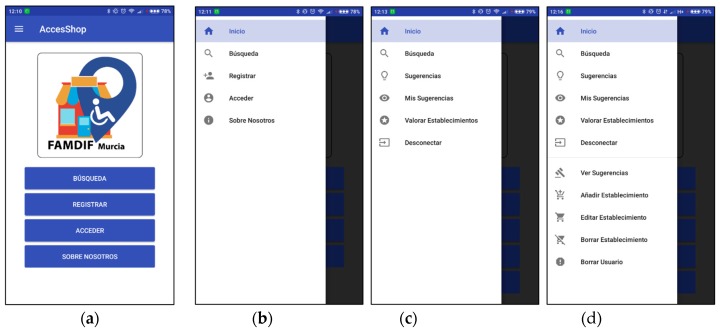
Different views of the app. (**a**) Initial screen. (**b**) Menu options for unregistered user. (**c**) Menu options for registered user. (**d**) Menu options for administrator user.

This section presents the implementation of the most interesting operation of the app, which is the search for accessible shops. Figure 6a shows the initial screen, which consists of a logo and several buttons that provide access to the available operations. The collapse menu button, or hamburger button, is placed in the top-left corner to allow the user to access the operations from any screen.

**Figure 7:**
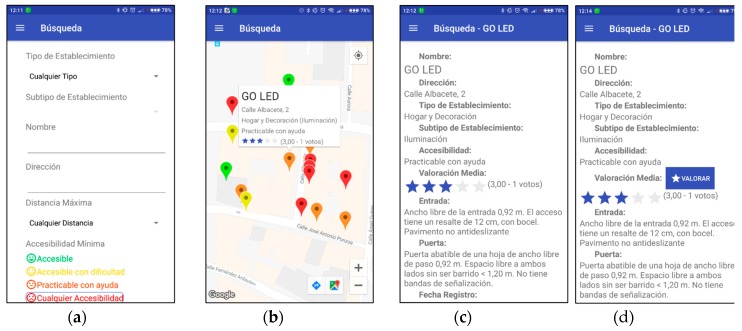
Different views of the app related to the search. (**a**) Search screen. (**b**) Search results after selecting a mark. (**c**) Shop details for an unregistered user. (**d**) Shop details for a registered user.

Once the menu is displayed, it is possible to access the different actions that can be performed depending on whether the user has logged in the application or not, and the role of the user (see Figure 6b–d). It can be observed that all users have access to the search operation (“Búsqueda” in Spanish). Figure 7a shows the screen which is opened after selecting this option; the search filters are displayed and can be selected or not by the user. The levels of accessibility are represented with standard colors: A—green; B—yellow; C—orange; D—red.

The shop filtering during the search process is done in different phases with the aim of reducing the use of data and energy of the app:

- *First filtering*. The app connects to the server indicating the search criteria. The records that contain the name, address, type, sub-type and level of accessibility queried by the user are obtained from the database, and the server returns a list of shops that meet these criteria, avoiding those that do not match them. Only the ids and GPS coordinates are sent, thus requiring a minimum amount of data.
- *Second filtering*. According to the current GPS location, the app calculates which shops are inside the maximum distance requested by the user; those that are at a greater distance are discarded. Then the app sends to the server the list of desired ids, and the server reports the list of shops including their basic characteristics: id, location, name, address, type, sub-type, level of accessibility, and evaluation. These are shown as color markers in a map; as depicted in Figure 7b, the markers are selectable showing the basic features of the shop.
- *Search details*. In the case that the user wants to see more detailed information about a specific shop, the id is sent to the server and it reports all the information stored in the database concerning this shop. It is shown in the app, as in Figure 7c. Additionally, the registered users have the possibility to rate the shops, Figure 7d.

### 4.2. Usability Evaluation

The needs of users have expanded further concerning mobile apps. In this sense, customers are the ones judging the usefulness of applications, which leads to a range of learnability attributes. In addition, usability in apps is closely related to learnability [23]. To this end, a usability evaluation of the app was conducted to assure a good reception among potential users.

There are three categories of usability evaluation methods [24]: inquiry, inspection, and testing. The usability inspection approach is carried out by usability specialists, and sometimes software developers, users and other professionals, who evaluate user interface elements against a set of specific criteria [25]. Some common inspection methods are heuristic evaluation [26] and cognitive walkthrough [27]. Both techniques help find obvious usability issues in a quick and inexpensive way [25] and can be applied at the deployment stage of the software development lifecycle [24].

Cognitive walkthrough was part of the method selected to examine usability in the app in order to provide depth and granularity to our evaluation process [28]. This technique allows us to evaluate the tool in a way a novice user would do, thus, people outside the development team carried out the analysis. A set of assumptions were proposed to refine the usability audit based on this technique:

- *Informativeness*. The information exposed in the application is positively associated with potential users.
- *Cognitiveness*. The cognitive skills of an individual will contribute more toward performing the task successfully over the knowledge of the same individual.
- *Ease of use*. Tech-savvy customers will navigate the application with ease when comparing to others.
- *Texts captions*. Length of the text is directly proportional to the informativeness quotient.
- *Integration*. By integrating cognitiveness principle with usability criteria, improvement in the expediency quotient is anticipated.

A brief questionnaire was proposed guided by the aforementioned assumptions. Evaluators answered the questions using a Likert-type scale ranging from “5” to “0”, where “5” means totally agreement, and “1” is totally disagreement. The score of “0” is interpreted as not having elements of judgment to express someone’s opinion. The results are shown in Table 2.

Another part of the usability audit was accomplished by means of a methodology based on expert review or heuristic evaluation, which is the most commonly used among usability inspection methods [29]. Numerous sets of heuristics can be applied during heuristic evaluation [24]; in our case, the usability principles by Dix et al. [30] were employed after the cognitive walkthrough process to complete the usability audit. Once all the activities in the application were reviewed, the usability principles by Dix were scored in a Likert-type scale. The usability principles by Dix are presented hereafter, and the results of the evaluation are depicted in Table 3:

- *Predictability*. Support for the user to determine the effect of future action based on past interaction history.
- *Synthesizability*. Support for the user to assess the effect of past operations on the current state.
- *Familiarity*. The extent to which a user’s knowledge and experience in other real-world or computer-based domains can be applied when interacting with a new system.
- *Generalizability*. Support for the user to extend knowledge of specific interaction within and across applications to other similar situations.
- *Consistency*. Likeness in input-output behavior arising from similar situations or similar task objectives.
- *Dialog initiative*. Allowing the user freedom from artificial constraints on the input dialog imposed by the system.
- *Multi-threading*. Ability of the system to support user interaction pertaining to more than one task at a time.
- *Task migratability*. The ability to pass control for the execution of a given task so that it becomes either internalized by the user or the system or shared between them.
- *Substitutivity*. Allowing equivalent values of input and output to be arbitrarily substituted for each other.
- *Customizability*. Modifiability of the user interface by the user or the system.
- *Observability*. Ability of the user to evaluate the internal state of the system from its perceivable representation.
- *Recoverability*. Ability of the user to take corrective action once an error has been recognized.
- *Responsiveness*. How the user perceives the rate of communication with the system.
- *Task conformance*. The degree to which the system services support all of the tasks the user wishes to perform, and in the way that the user understands them.

Given that observers will sometimes agree or disagree simply by chance, it is necessary to measure the agreement between raters using a statistic, and in this regard the kappa coefficient is the most common [31]. Since we had to deal with ordered-category data and two observers, weighted kappa with Fleiss-Cohen (quadratic) weights was applied in this study [32]. The interrater reliability value was found to be kappa = 0.6673, 95% CI (0.3649, 0.9697), which accounts for a *substantial* level of agreement [33].

## 5. Discussion

The development of this app shows the relationship between architecture and new technologies, and also the importance of new technologies for people with disabilities in their daily life. In this case, the app, called *ACCEDE Murcia*, offers the possibility to have information about the shops of the city of Murcia. It is endowed with especially important characteristics for people with disabilities such as: easy to use, low use of data and battery, capability to adapt the app to English or Spanish depending on the language of the mobile phone. We have not found any app considering i18n requirements [34].

Comparing to other existing apps, most of the apps found in the mobile platforms (*Access GC*, *Access Aware*, *Access Earth*, *Accessibility Plus* and *Mapcesible*) provide information about disability carparks. The lack of accessible car parks is one of the main barriers restricting independent travel for people with disabilities [35]. Other disability services such as accessibility to restaurants (*Access GC*, *Access4All*, *AccessAble*, *AccessNow*, *Accessibility Plus* and *Mapcesible*), toilets (*Access GC*, *AccessAble* and *AccessNow*) and hotels (*Access GC*, *Access Earth*, *AccessAble*, *AccessNow*, *Access4All*, *Accessibility Plus* and *Mapcesible*) are also very common. Empirical studies suggest that accessibility to public areas and bath in rooms are two of the strongest predictors of the hotel satisfaction for people with physical disabilities [36].

All the analyzed apps provide users with the possibility of opening Google Maps. The presented *ACCEDE Murcia* includes also this feature to enable computing a route to the selected establishment. However, the lack of detailed information about features of the route such as sidewalks, surface conditions or road incline, prevents developers from obtaining friendly routing networks for people with disabilities. These prerequisites of special consideration should be taken into account so routing applications are tailored for them. Algorithms using collaboratively collected geodata to generate routes for people with disabilities have been proposed in literature [37].

Our app follows the Spanish regulation considering security of use and accessibility in buildings. Notice that some apps are dedicated to enforcement of accessibility laws such as parking misuse (*Access Aware*). Other apps focus on offering a checklist to assess common building elements in facilities. In particular, *Access Inspector* includes a checklist to assess more than 40 common architectural elements to create accessible environments such as: access routes, kerb ramps, entrances, doors, corridors, ramps, toilets, elevators and signage. Some apps such as *Accessibility Plus* and *Mapcesible* allow reporting new points of interest to the system. These functionalities may be an important tool to accessibility audit in commercial complexes, since limited guidelines and weak enforcement by the authorities have been identified in the literature [38].

The proposed system of suggestions of *ACCEDE Murcia* lets the user report and communicate with FAMDIF through the evaluation scores. Users can rate places by their accessibility in most of the apps studied (*Access Earth*, *Access Aware*, *AccessAble*, *AccessNow*, *Access4All*, *Accessibility Plus* and *Mapcesible*) which are then available to everyone. Rating provides both qualitative and quantitative data about the perception of people with disabilities of the amenities and places. The system of vote of *ACCEDE Murcia* has been designed to encourage the establishments with defaults to improve their accessibility. Previous studies investigating the relationship between rating and popularity have shown that apps that achieve a higher rating also tend to be more popular [39]. Therefore, high user ratings and good reviews might have a positive impact on the sales of the establishments. However, since consumers increasingly trust in user reviews and ratings, fraudulent reviews can be created to increase sales and damage competitors’ reputations [40]. The apps should control bad intentioned users. For example, a study published by Harvard Business School reported that 20% of all online restaurant reviews on Yelp are fake [41]. This can also degrade user rely on online reviews, which is a promising topic for future research. Notice that only our app, *ACCEDE Murcia* (FAMDIF), *Access GC* (Open data) and *Access4All* (Sapphire Support Services and Bureau of Accessible Tourism) use as reliable data sources to evaluate access for people with disabilities. The rest of apps employ citizens or users to measure the degree of accessibility.

## 6. Conclusions and Future Work

The proposed app has been developed to meet the requirements of sustainability, in both aspects of sustainable *in* software and *by* software. The app provides people with disabilities with an easy way to search and filter the establishments. The results are shown in a graphic, visual and intuitive way to give to the user the power to choose the establishment which is more adequate to their requirements. In this sense, some authors have indicated that the filtering process is a key element in applications for people with a disability [42]. Furthermore, the app will not only be beneficial for users, but will also help improve awareness of accessibility in local businesses.

The results of the usability evaluation suggest that the app offers a pleasant user experience (UX). A set of features should, however, be taken into account to make the UX more complete. Firstly, the *customizability* attribute of the app could be improved to offer more adaptation to the personal preferences of the users (e.g., we could add personal profile support to allow the user to customize for example the color schema, font size, etc.). Secondly, in relation to the *substitutivity* usability principle, the pictures of the shops could be included, since they can visually clarify the ease of entrance and movement inside the facilities, but this feature should be widely accepted and implemented by the businesses’ owners. Thirdly, *multi-threading* could be enhanced by managing several information sources (e.g., textual data about the shops, maps, photos) at the same time.

Regarding the future research lines of this project, we consider several aspects which could be included in the development of the app:

- Access to the app through social networks like Facebook. Successful social inclusion can be achieved by being part of a social network of people in the community [43]. In addition to this inherent benefit for people with disabilities, users’ opinion can have an effect on compliance with regulations by the establishments. Empirical studies have shown that opinions in online social networks play an important role in the information diffusion [44]. On the other hand, social network analysis can be used to capture the structural features such as junctions and routes, with the aim of assessing accessibility and providing connections to establishments [45]. Moreover, online social networks are a great showcase for owners, so providing accessible environments can open new markets and business opportunities to owners.
- Inclusion of information related to the user such as age, sex and type of disability, with the aim of making a statistical study about the users of the app and produce a report of searches to know the information which is looked for by the users. Aggregated statistical reporting on users registered will be prepared for disability support, to help administrators manage and improve the functionality provided by the app. However, a privacy policy should be defined [46,47] based on “Privacy by Design” [48] to be considered throughout the whole app lifecycle. Data controllers and processors must be proactive in addressing the privacy implications of any new change in the system. Notice that sensitive personal information is a special category and will only be available under explicit consent. Therefore, people with disabilities must be in control of who they tell about their disability. Information without their permission must not be passed on. Access to personal information will be limited by system permissions to authorized people to comply with the Equality Act 2010. Although electronic consent (eConsent) has not been widely used [49], an eConsent process might be implemented to adopt a more visual approach with voice over capabilities [50], thus being more accessible to users with disabilities.
- Add photos of the shops. We intend to include photos of the establishments to show in a visual way the entrance, door and all the information stored about the establishment. This feature is usually included in the apps for people with disabilities. In addition, Google Maps® can provide 360º pictures of the shops interior to show the distribution of the products. Textual information might not reflect important details which are shown in an image. Moreover, studies have reported that individuals can categorize visual information faster than using words [51]. These features could make it easier for people with disabilities to identify the access that best suit their needs.

## Figures and Tables

**Table 1:** Summary of the levels of accessibility to the buildings defined in this research.

Code	Name	Characteristics
A	Accessible	Access at street level, or through a ramp with maximum slope of 8%, or a step up to 1 cm.
B	Accessible with difficulty	Access with a ramp of 10% slope or less, or a step up to 5 cm overcome with a ramp of 25% slope or less.
C	Practicable with help	Access with a ramp with 12% slope or less, or a step up to 12 cm alone or with a ramp of 30% slope or less, or a step up to 3 cm round or beveled.
D	Not accessible	The rest of the cases.

**Table 2:** Cognitive walkthrough evaluation of the app *ACCEDE* Murcia.

Question	Evaluator A	Evaluator B
Did you find the shop of your interest?	4	4
Did you find shop alternatives?	3	4
Were the entrance of the shop as it appears in the app?	4	4
Were you able to find other information regarding the shop?	5	4
Was it easy to find a shop in the app?	4	4
Was it comfortable to search for information in the app?	3	4
Did you find all the information you need concerning access to the shop?	2	3
Were you able to register your profile?	4	5
Were you able to send suggestions?	4	5

**Table 3:** Usability principles by Dix usability evaluation.

Usability Principles by Dix	Evaluator A	Evaluator B
Predictability	5	5
Synthesizability	4	4
Familiarity	4	4
Generalizability	4	5
Consistency	5	4
Dialog initiative	4	3
Multi-threading	2	3
Task migratability	3	3
Substitutivity	2	2
Customizability	1	1
Observability	3	4
Recoverability	4	4
Responsiveness	4	5
Task conformance	5	3
